# Scope of claim coverage in patents of *fufang *Chinese herbal drugs: Substitution of ingredients

**DOI:** 10.1186/1749-8546-6-30

**Published:** 2011-08-19

**Authors:** Xinsheng Wang, Jiaher Tian, Albert Wai-Kit Chan

**Affiliations:** 1 DeHeng Chen, LLC, 225 Broadway, Suite 1910, New York, NY 10007, USA; 2 Forest Laboratories, Inc., 220 Sea Lane, Farmingdale, NY 11735, USA; 3 Law Offices of Albert Wai-Kit Chan, PLLC, Whitestone, New York 11357, USA

## Abstract

Herbal ingredients in a Chinese *fufang *prescription are often replaced by one or several other herbal combinations. As there have been very few Chinese herbal patent infringement cases, it is still unclear how the Doctrine of Equivalents should be applied to determine the scope of 'equivalents' in Chinese *fufang *prescriptions. Case law principles from cases in other technical areas such as chemical patents and biological drug patents can be borrowed to ascertain a precise scope of a *fufang *patent. This article summarizes and discusses several chemical and biopharmaceutical patent cases. In cases where a certain herbal ingredient is substituted by another herb or a combination of herbs, accused infringers are likely to relate herbal drug patents to chemical drug patents with strict interpretation whereas patent owners may take advantage of the liberal application of Doctrine of Equivalence in biopharmaceutical patents by analogizing the complex nature of herbal drugs with biological drugs. Therefore, consideration should be given to the purpose of an ingredient in a patent, the qualities when combined with the other ingredients and the intended function. The scope of equivalents also depends on the stage of the prior art. Moreover, it is desirable to disclose any potential substitutes when drafting the application. Claims should be drafted in such a way that all foreseeable modifications are encompassed for the protection of the patent owner's intellectual property.

## Introduction

In Chinese medicine practice, single herbal ingredient prescriptions are referred to as *danfang *whereas multiple herbal ingredients prescriptions are *fufang *which is more widely used than *danfang *due to the synergistic effects. Many herbal ingredients in a *fufang *prescription may be replaced by one or several other herbal combinations without failing to produce similar therapeutic effects. For example, *Rhizoma Coptidis *(*Huanglian*), *Cortex Phellodendri *(*Huangbai*) and *Radix Scutellariae Baicalensis *(*Huangqin*) share similar functions and may be replaced with each other for clearing *heat*, drying *dampness*, draining *fire *and relieving *toxicity*. Ginseng, when taken orally as adaptogen, aphrodisiac and nourishing stimulant, may be substituted by other herbs in the ginseng family, such as *Dangshen *and *Huangqi*.

The existence of multiple substitutes often makes patent owners concerned about limiting the scope of their claims to a particular herbal combination. For example, if someone obtains a *fufang *patent X comprising A, B, C and D, does a composition consisting of A, B, C and P, or a composition consisting of A, B, C, E and F infringe patent X? The answer to this hypothetical question depends on how the court interprets the scope of the claim coverage in a *fufang *patent.

## Claim interpretation in general

To ascertain the precise scope of a patent, one must look at (1) the literal scope of a patent claim and (2) the scope of claim coverage under the Doctrine of Equivalents. Claim construction analysis begins with the literal words of the claim, which generally carries their ordinary and customary meanings [1]. The claims 'must be read in view of the specification, of which they are a part' [2].

The Doctrine of Equivalents extends the patentee's right to exclude others from making, using, selling or importing the patented invention beyond the literal scope of the claims. Infringement under the Doctrine of Equivalents is an equitable doctrine devised for 'situations where there is no literal infringement but [where] liability is nevertheless appropriate to prevent what is in essence a pirating of the patentee's invention' [3].

The principal limitation on the scope of equivalents to which a patentee may be entitled is prosecution-history estoppel. The essence of prosecution-history estoppel is that patent owners may not recapture through litigation any subject matter they previously surrendered during prosecution. Prosecution-history estoppel prevents patent owners from using the doctrine of equivalents during litigation to argue that the scope of a patent claim should be interpreted to include subject matter surrendered in a narrowing amendment or implicitly by argument [4].

After remand from the Supreme Court construed the Supreme Court's decision in *Festo VIII*, the Federal Circuit's decision in *Festo Corp*. (*Festo IX*) [5] held that (1) 'a narrowing amendment made to satisfy any requirement of the Patent Act may give rise to an estoppel' and (2) that there is a presumption that a narrowing amendment made for a reason of patentability surrenders the entire territory between the original claim limitation and the amended claim limitation [6]. A patentee may overcome that presumption by showing that at the time of the amendment one skilled in the art could not reasonably be expected to have drafted a claim that would have literally encompassed the alleged equivalent [7].

## Application of the Doctrine of Equivalents in *fufang *patents

For *fufang *patents, how the Doctrine of Equivalents will be applied to determine the scope of an 'equivalents' is still unclear. The court has received very few Chinese herbal patent infringement cases. As such, one must borrow case law principles from cases in other technical areas. The most related technical area probably is chemical drug patents, where the Doctrine of Equivalents has matured. The next related technical area is biological drug patents. For the purpose of this article, a few chemical and biotechnological patent cases are summarized and discussed help the readers understand how the Doctrine of Equivalents could be applied to Chinese herbal patents.

## Chemical drug patent cases

Chemical drug patents are often interpreted strictly. A charge of infringement would likely be avoided by any addition, elimination of an active ingredient, or a change in their proportions [8]. The courts often find non-infringement if the ingredient being substituted is new, performs a substantially different function or is not known at the date of plaintiff's patent as a proper substitute for the one omitted.

One illustrative example is *Parmlee Pharmaceutical Co. v. Zink*, which involves United States Patent No. 2,478,182 issued to William V. Consolazio [9]. Parmlee Pharmaceutical is the exclusive licensee of the patent. There is only one claim in the patent as follows:

'An internally reinforced sodium chloride tablet comprising compressed granules of sodium chloride; and an internally disposed cellular stroma of a thin, permeable, dialyzing film of a material selected from the group consisting of cellulose acetate and cellulose nitrate, the cells of said stroma containing said granules of sodium chloride, whereby the sodium chloride is rendered slowly available when the tablet reaches the gastro-intestinal tract, the solution time of the sodium chloride in said tablet in the gastrointestinal fluids being from 60 to 80 minutes for a ten grain tablet.'

The specification of the patent refers to the use of salt tablets to combat the ill effects of heat and excessive sweating. Ingestion of a salt tablet is frequently associated with incidence of epigastric discomfort, nausea and vomiting. A popular theory at the time of the invention was that quick absorption of the salt tablet would shorten the irritation time to the gastro-intestinal tract. The patentee's innovative concept, however, is that a slow absorption rate of salt would eliminate the discomfort in gastro-intestinal tract entirely. The slow-release result was accomplished by coating the salt tablet with a cellular stroma of a film made from cellulose acetate or cellulose nitrate.

The plaintiff's product became a commercial success. The accused tablet conformed in general construction to the patentee's tablet and accomplished the same result. The film employed in the accused infringer's tablet, however, was not of 'the group consisting of cellulose acetate and cellulose nitrate'. The film was, instead, 'shellac'.

The accused product is not a literal infringement of the '182 patent, because the patent's claim was limited to tablets coated with cellulose acetate or cellulose nitrate'. The plaintiffs argued that the accused tablets were equivalents and reasoned that Consolazio's approach to controlled release was radically different from that of the past art, and accordingly, the patent was entitled to a broad range of equivalents which would embrace *every *material which might be used to form the particular structure, *ie *a thin permeable dialyzing film disclosed in the patent.

The defendants argued that numerous prior art had taught what materials could be applied to make the coating and the use of the word 'consisting' in the claim and its reference to cellulose acetate and cellulose nitrate was restrictive and operated to exclude all substances not belonging to that group. The Federal Circuit agreed with the defendants that while the coatings performed the same function, so many different coatings were known prior to the patent's filing that it would be inequitable to expand the scope of the claim beyond the two listed substances. In the light of the prior art, the court held that 'the proper range of equivalents for this patent is a narrow one and ... is of insufficient breadth to include a substance' other than the two recited in the claim [9].

**Figure 1:**
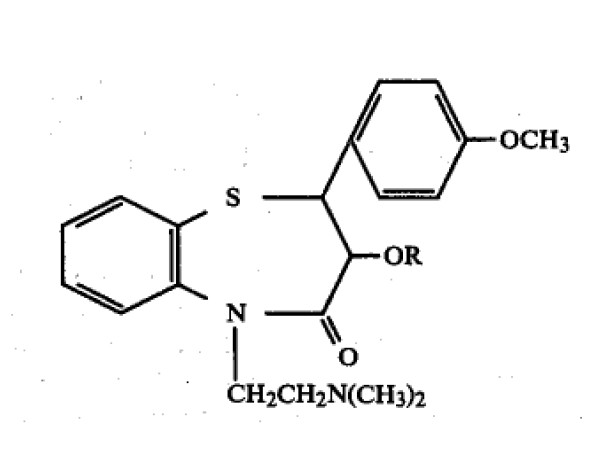
**The chemical structure of benzothiazepine derivative in claim 1 of the '035 patent**.

**Figure 2:**
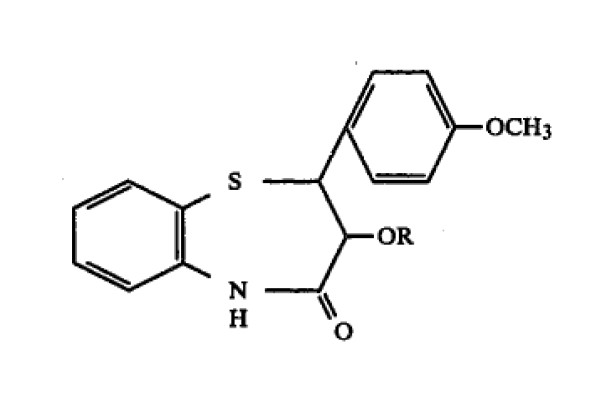
**The chemical structure of the compound for preparing benzothiazepine derivative in claim 1 of the '035 patent**.

In another case, *Tanabe Seiyaku Co., Ltd. v. U.S. International Trade Commission*, the question of whether acetone (CH_3_C( = O)CH_3_) was equivalent to butanone (CH_3_C( = O)CH_2_CH_3_) as a solvent for a chemical reaction was argued before the court [10]. The plaintiff's patent No. 4, 438, 035 was a process patent claiming a chemical process for preparing diltiazem hydrochloride, a pharmaceutical product used to treat various cardiovascular diseases. Claim 1 of the '035 patent reads: 'A method of preparing a benzothiazepine derivative of the formula (Figure 1) wherein R is a hydrogen or acetyl, or a pharmaceutically acceptable acid addition salt thereof, which comprises condensing a compound of the formula (Figure 2) wherein R is the same as defined above, with 2-(dimethylamino)ethyl halide either in the presence of potassium hydroxide in acetone or in the presence of potassium carbonate in a solvent selected from acetone, lower alkyl acetate, a mixture of acetone and water and a mixture of lower alkyl acetate and water, and if required, further converting the product into a pharmaceutically acceptable acid addition salt thereof.'

The chemical reaction recited in claim 1 is known as an 'N-alkylation' reaction. The five base-solvent combinations disclosed in the '035 patent and recited in claim 1 are listed in Table 1. The accused Fermion process involved performing the N-alkylation reaction in the presence of the base potassium carbonate and, rather than acetone, the solvent butanone mixed with water. As the parties agreed that the use of this base-solvent combination was not within the literal language of claim 1, the issue became whether the use of butanone in the Fermion process instead of the acetone in the patent claim constituted a 'substantial' or 'insubstantial' difference between the accused process and the patent.

**Table 1:** Five base-solvent combinations disclosed in the '035 patent

Combination	Base	Solvent
1	potassium hydroxide	acetone
2	potassium carbonate	acetone
3	potassium carbonate	acetone and water
4	potassium carbonate	lower alkyl acetate
5	potassium carbonate	lower alkyl acetate and water

The defendant presented evidence that duplicating examples from the patent with butanone instead of acetone often gave poor results, while in one case, the result with butanone was better. A good deal of experimentation was performed by the defendant while optimizing a scaled-up reaction, from which the court inferred that the defendant had designed around, rather than copied, the patented method, and that butanone and acetone were not truly interchangeable.

## Biological drug patent cases

While chemical drugs are small molecules mostly produced by chemical synthesis, biological drugs are macromolecules consisting of thousands of atoms. They are made by living cells (bacteria, yeast, animal or human cells) whose DNA has been modified by introduction of the gene of interest to synthesize the active component. The complex structures of biological drugs are often composed of multiple long chains of amino acids, derivatized by sugar moieties and folded by complex mechanisms.

Due to its complexity, biological drugs cannot be fully copied. No two cell lines, developed independently, can be considered identical. A multiplicity of DNA sequences encodes the same protein. One or several amino acid additions, substitutions or deletions can be made in one protein sequences with retention of the desired activity. As a consequence, the term 'biosimilar' is used to acknowledge the fact that while biosimilar products are similar to the original product, they are not exactly the same.

Compared to the chemical drug patents, claims for biological drug patents are interpreted more broadly. The courts have applied the Doctrine of Equivalents more liberally [11]. When deciding whether the accused drug is a 'functional equivalent' of the patented drug, 'similarities' between the accused drug with the patented item are compared, rather than structural identity. Does the accused drug exert the same therapeutic effects? Are the structures of the active residuals identical? Does the accused drug trigger the same immunogenic response? A sequence with a silent nucleotide substitution, *ie *one that produces no alteration in the amino acids sequence of the expressed protein, will always be an infringement under the Doctrine of Equivalents [12].

On the other hand, if the difference between the patented compound and the accused compound is substantial and significant, a finding of equivalents might not sustain [13]. In *Genentech, Inc. v. The Wellcome Foundation, Ltd*., Genentech owned three patents, namely a patent directed to a natural protein extracted from certain human cancer cells, a patent directed to the materials needed to produce the natural protein through recombinant DNA technology and the microorganism or cell culture capable of expressing the protein. The Wellcome Foundation produced a protein through recombinant DNA technology. The accused version of fPA differred by one amino acid in a kringle region, with a half-life ten times of natural t-PA. Nevertheless, the accused protein exhibited the same clot dissolving activity of tPA. A jury at the trial court held that the accused version infringed Genentch's patent under the Doctrine of Equivalents, while concluding that they did not fall literally within the scope of Genentech's patent. The district court holding was reversed by the Federal Circuit [14]. The court held that a 15% difference of the amino acids sequnce between the accused protein and the claims are substantial. More importantly, the accused proteins did not bind to fibrin in a manner equivalent to native tPA [14].

Nevertheless, given the impact of the *Festo IX *[6] owners of biological patents could end up with undesirable claim scope due to untactful claim amendment during patent prosecution. For any narrowing amendment made after Festo, presumption is that a narrowing amendment, made for any reasons related to patentability, raises a rebuttable presumption that infringement under the doctrine of equivalents is not available for subject matter surrendered by the narrowing amendment. To rebut the presumption that estoppel applies, the patentee must show that either (1) the equivalent was 'unforeseeable at the time of the application'; (2) 'the rationale underlying the amendment [bears] no more than a tangential relation to the equivalent in question' or (3) 'some other reason suggest[s] that the patentee could not reasonably be expected to have described the insubstantial substitute in question'[7]. Therefore, a variety of claims presenting differing narrowing limitations should be included at the time of drafting the application. Applicants should avoid presenting overly broad claims if the broad claims would likely be amended during the examination process. Otherwise, the patent owner may end up with a much narrower patent which may not be enforceable against a competitor because of a Festo-type estoppel based on the narrowing amendment.

For example, in *Schwarz Pharma v. Paddock, Schwarz Pharma *owns U.S. Patent 4,743,450 ('the '450 patent') [15]. The '450 patent was entitled 'Stabilized Compositions' relating to pharmaceutical compositions containing Angiotensin Converting Enzyme (ACE) inhibitors for the treatment of hypertension. The ACE inhibitors must be combined with stabilizers to avoid degradation, discoloration and hydrolysis. The '450 patent generally teaches the use of an alkali or alkaline earth metal carbonate to inhibit cyclization and discoloration. Claims 1 of the patent reads: 'A pharmaceutical composition which contains: (a) a drug component which comprises a suitable amount of an ACE inhibitor which is susceptible to cyclization, hydrolysis, and discoloration, (b) a suitable amount of an alkali or alkaline earth metal carbonate to inhibit cyclization and discoloration, and (c) a suitable amount of a saccharide to inhibit hydrolysis' [15].

*Paddock *filed abbreviated new drug application (ANDA) for generic moexipril formulation, using magnesium oxide (MgO) as a stabilizer. *Schwarz Pharma *sued *Paddock *for patent infringement, arguing that MgO is a foreseeable equivalent of magnesium carbonate described in patent. The District Court of Minnesota found non-infringement based on prosecution history estoppel. Originally, the independent Claim 1 recited 'an alkali or alkaline earth-metal salt'; however, it was amended to instead recite 'an alkali or alkaline earth metal carbonate' following the rejection. The court held that the change in claim language was a narrowing amendment and presumptively surrendered all metal containing stabilizers and alkali or alkaline earth metal salts except alkali and alkaline earth metal carbonates. The court also held that *Schwarz *had failed to rebut the presumption of surrender because magnesium oxide was a foreseeable equivalent of magnesium carbonate and because there was no objectively apparent reason for the narrowing amendment not directly related to the use of magnesium oxide. The court thus concluded that the Paddock drug did not infringe because *Schwarz *was estopped from claiming that the magnesium oxide used by Paddock was the equivalent of an alkali or alkaline earth metal carbonate. The decision was later confirmed by the Federal Circuit.

## Chinese herb patents

As very few herbal patent infringement cases have been decided by the court, how the Doctrine of Equivalents will be applied in herbal patents is unclear. Both patent owners and the accused infringers would have to refer to case law principles from chemical and/or biological patent cases.

As the active ingredients identified in herbal medicines are mostly small molecules, it is reasonable that herbal patents should follow the general rule for chemical drugs. The accused infringers are likely to argue that the substituted herbal ingredient adds new chemical compounds into the drug composition, thereby performing different functions. Herbal drug patent owners, nevertheless, may argue that herbal drugs are very different from chemical drugs due to the complex nature of herbal drugs. Herbal drugs often contain multiple active ingredients; furthermore, a *fufang *herbal drug often includes over a dozen herbs. The complexity of an herbal drug is further enhanced by the production process. As such, every herbal drug can be considered unique and this is very similar to biological drugs. Substituting or changing the amount of a minor herbal component in a *fufang *drug will lead to a 'different' composition but the change may or may not cause difference in the overall therapeutic effect. Therefore, 'similarities' rather than 'identity' should be compared. Unless the 'similarities' of the herbal drugs are changed, the substitute should be considered as an 'equivalent' and within the scope of the claims.

For example, substitution of a principal herbal ingredient in a *fufang *drug may alter its therapeutic effects and potential side effects. The original patented *fufang *composition and the substituted *fufang *should not be considered as 'equivalents'. However, minor distinctions (*eg *ratio of composites, manufacturing process) should be recognized under these circumstances. Another important factor is whether persons reasonably skilled in the art would have known of the interchangeability of the substituting ingredient [9].

The best time to protect the patent owner's intellectual property is at the time of drafting the application. While it is impossible to cover all possible equivalents, it is advisable to disclose any potential substitutes. Claims should be drafted to literally encompass all foreseeable modifications. The specification should also disclose any 'functional equivalents' at the time of drafting. The applicant must define exactly which variations may be included, possibly based on some experimental result and clinical data [16].

## Concluding remarks

In drafting a patent application for a Chinese herbal drug, one should give consideration to the intended purpose of an ingredient, the quality when combined with the other ingredients and the intended function. What constitutes an equivalent must be determined against the context of the patent, the prior art and the particular circumstances of the case. The range of equivalents is broader in an uncrowded art and narrower in a crowded one.

## Competing interests

The authors declare that they have no competing interests.

## Authors' contributions

AWKC conceived the study and revised the manuscript. XW and JT researched on the subject and drafted the manuscript. All authors read and approved the final version of the manuscript.
